# Effects of Phrenic Nerve Stimulation in Mechanically Ventilated Patients: A Systematic Review and Meta-Analysis of Randomized Controlled Trials

**DOI:** 10.3390/jcm15114245

**Published:** 2026-05-30

**Authors:** Xi Wang, Hao Dong, Qi Ren, Qianghong Xu

**Affiliations:** Department of Critical Care Medicine, Zhejiang Hospital, No. 1229, Hangzhou 310030, China; wang210xi@163.com (X.W.); wujieping1917@163.com (Q.R.); xqhong@163.com (Q.X.)

**Keywords:** phrenic nerve stimulation, mechanical ventilation, ventilator-induced diaphragmatic dysfunction, weaning

## Abstract

**Objectives:** This systematic review and meta-analysis aimed to evaluate the efficacy and safety of phrenic nerve stimulation (PNS) in mechanically ventilated adult patients. **Methods:** PubMed, Web of Science, Embase, and the Cochrane Library were searched for randomized controlled trials (RCTs) up to 21 February 2026, without language restrictions. Two reviewers independently screened studies, extracted data, and evaluated the risk of bias using the Cochrane RoB 2 tool. The certainty of evidence for each outcome was assessed using the Grading of Recommendations Assessment, Development, and Evaluation (GRADE) framework. Odds ratios (ORs) and mean differences (MDs) with 95% confidence intervals (CIs) were pooled using fixed-effects or random-effects models according to heterogeneity. **Results:** Five RCTs involving 431 patients were included. PNS was associated with a higher weaning success rate (OR = 2.96, 95% CI: 1.04 to 8.40, *p* = 0.04), a shorter duration of mechanical ventilation (MD = −2.63, 95% CI: −3.90 to −1.35, *p* < 0.001), higher maximal inspiratory pressure (MD = 2.95, 95% CI: 1.10 to 4.79, *p* = 0.002), and higher diaphragm thickening fraction (MD = 15.67, 95% CI: 4.84 to 26.50, *p* = 0.005). No statistically significant differences were observed in ICU length of stay, rapid shallow breathing index, or tracheostomy rate. Noninvasive stimulation was generally tolerated in the included studies, whereas transvenous stimulation was associated with procedure-related serious adverse events. The certainty of evidence ranged from high to low across outcomes. **Conclusions:** PNS was associated with improved weaning outcomes and diaphragm function in mechanically ventilated patients. However, the evidence remains limited by the small number of RCTs, clinical heterogeneity, and uncertainty regarding long-term outcomes. Further large-scale, multicenter RCTs with standardized protocols are needed to assess the efficacy and safety of PNS.

## 1. Introduction

Mechanical ventilation (MV) is a core life-support technology for the management of critically ill patients in the intensive care unit (ICU) [1]. It is widely applied in the management of acute respiratory failure and acute respiratory distress syndrome (ARDS) [2]. By maintaining effective gas exchange and reducing the load on respiratory muscles, MV provides critical time for the treatment of the primary disease [3]. However, MV is also associated with complications, among which the early development of ventilator-induced diaphragmatic dysfunction (VIDD), which can emerge within the first 24 h of controlled ventilation, represents a major clinical challenge [4]. VIDD, characterized by diaphragmatic atrophy and weakness, is a key factor leading to difficult weaning. Upon weaning failure, patients are at increased risk of prolonged mechanical ventilation (PMV), which can increase the incidence of complications such as ventilator-associated pneumonia (VAP) and airway injury. This cycle of VIDD, difficult weaning, and prolonged ventilation may prolong hospitalization, thereby increasing medical costs and mortality risk [5,6].

Accurate assessment of weaning readiness is therefore essential to avoid both premature extubation and unnecessary prolongation of MV. While artificial intelligence (AI) and machine-learning models have recently been explored as decision-support tools for predicting weaning success in patients with respiratory failure, including those with ARDS [7], prediction alone cannot prevent or reverse diaphragm unloading and VIDD. Therefore, active diaphragm-protective interventions remain necessary. Traditional clinical strategies for the prevention and treatment of VIDD have predominantly focused on passive protection, including low tidal volume and other lung-protective ventilation strategies [8]. However, these methods primarily mitigate the risk of diaphragmatic injury and do not actively maintain diaphragm activity. Furthermore, many critically ill patients, especially those requiring prolonged mechanical ventilation, typically cannot engage in traditional respiratory muscle training due to impaired consciousness and profound muscle weakness, which further limits the recovery of diaphragm function [9]. Therefore, there remains a need to develop a non-volitional, clinically feasible modality for diaphragmatic protection and rehabilitation.

In this context, phrenic nerve stimulation (PNS), also referred to as diaphragm neurostimulation, is considered an active intervention designed for the regulation of diaphragm function [10]. Its underlying mechanism involves the administration of low-frequency electrical pulses through external or implantable electrodes to selectively stimulate the phrenic nerve, thereby aiming to preserve the structural integrity of diaphragm fibers and improve diaphragmatic muscle strength and endurance [11].

PNS is broadly divided into two major technical systems, namely permanent diaphragmatic neurostimulation and temporary diaphragmatic neurostimulation [12]. The former is indicated for patients with chronic diseases who retain intact phrenic nerve function but lack central respiratory drive [e.g., patients with high-level spinal cord injury (SCI) or central sleep apnea (CSA)] [13,14]. It requires surgical implantation of electrodes to achieve long-term ventilator independence. Because of its surgical invasiveness and the need for postoperative recovery, it is not suitable for critically ill patients [15]. Conversely, temporary diaphragmatic neurostimulation targets patients with acute respiratory failure and critical illness. Its technical subtypes are primarily classified by anatomical approach and invasiveness into two main categories: transcutaneous stimulation and transvenous stimulation [12]. The former approach is noninvasive and utilizes surface electrodes. Its protocols vary according to electrode placement, that is, on the neck for phrenic nerve stimulation (e.g., external diaphragmatic pacing, EDP) or on the thorax (e.g., parasternal and intercostal) to directly stimulate the diaphragm (termed transcutaneous electrical diaphragmatic stimulation, TEDS) [16]. In contrast, the transvenous approach, known as temporary transvenous diaphragm neurostimulation (TTDN), is minimally invasive and utilizes a specialized central venous catheter with electrodes to indirectly stimulate the phrenic nerves through the wall of the superior vena cava. Additionally, subtypes such as percutaneous diaphragmatic stimulation, transcutaneous electromagnetic diaphragmatic stimulation, and transesophageal diaphragmatic stimulation are rarely applied in clinical practice due to limited technical maturity or insufficient clinical evidence [11,12].

In recent years, research investigating the clinical efficacy of PNS in mechanically ventilated patients has yielded inconsistent results [17,18,19,20,21]. On the one hand, several studies have reported favorable effects, with some studies reporting that noninvasive transcutaneous stimulation increased weaning success rates and shortened MV duration [17,18]. On the other hand, a study by Medrinal et al. concluded that TEDS failed to improve diaphragmatic function or muscle strength [19]. Furthermore, a recent large multicenter randomized controlled trial reported that TTDN demonstrated a high posterior probability of benefit regarding weaning success [21]. Amid this inconsistent body of evidence, existing meta-analyses also have limitations. First, most previous reviews have focused on chronic respiratory dysfunction populations, such as patients with CSA or high-level SCI, and rarely included ICU patients requiring MV [22,23,24]. Second, a recent meta-analysis that involved MV patients included both observational studies and RCTs. This approach may introduce heterogeneity. Indeed, its reported heterogeneity for MV duration was as high as *I*^2^ = 99% [25]. Given the discrepancies in primary research findings and the methodological limitations of existing meta-analyses, this systematic review and meta-analysis aims to evaluate the effectiveness and safety of PNS in mechanically ventilated patients, incorporating only RCTs.

## 2. Materials and Methods

### 2.1. Protocol and Registration

This systematic review and meta-analysis was conducted in accordance with the Preferred Reporting Items for Systematic reviews and Meta-analyses (PRISMA) guidelines [26] (Supplementary Table S1). The study protocol was prospectively registered on the international PROSPERO database for systematic reviews (CRD420251139751).

### 2.2. Eligibility Criteria

The inclusion and exclusion criteria for the primary studies were established based on the PICOS (Population, Intervention, Comparison, Outcomes, Study design) framework, as detailed in Table 1.

### 2.3. Search Strategies

A systematic literature search was conducted across PubMed, Embase, Web of Science, and the Cochrane Library from inception to 21 February 2026, without language restrictions. The search strategy employed a combination of Medical Subject Headings (MeSHs) and free-text terms, including keywords such as “electric stimulation”, “phrenic nerve”, “diaphragm”, “diaphragm neurostimulation” and “mechanical ventilation”. The full search strategy is detailed in Supplementary Table S2. Additionally, the reference lists of all relevant articles and citations within previously published meta-analyses were reviewed to identify additional eligible studies. Zotero (version 7.0) was used for literature management. After removing duplicates, two independent reviewers screened titles and abstracts. Full-text articles were assessed for inclusion independently, with discrepancies resolved by consensus with a third reviewer.

### 2.4. Data Extraction

Two authors independently extracted data from the included studies using standardized and piloted forms to maintain consistency. Prior to formal extraction, the forms were trialed on a subset of eligible studies. The two versions were compared, and discrepancies were resolved through discussion or adjudication by a third assessor. The following data were extracted from each study: (1) Study characteristics, including first author, publication year, region, and study design; (2) Participant characteristics, including the number of participants, age, and MV duration; (3) Intervention details (device, settings, stimulation site, and duration) and control methods; (4) The primary outcome measure was the weaning success rate, defined as the proportion of patients who successfully extubated and maintained spontaneous breathing for more than 48 h without reintubation [27]. The secondary outcome measures included: (1) mechanical ventilation duration (DMV): the time from the start of mechanical ventilation to successful extubation; (2) maximal inspiratory pressure (MIP): the maximum pressure generated by the patient’s voluntary inspiratory effort, measured at the end of the endotracheal tube with a pressure gauge [28]; (3) diaphragm thickening fraction (DTF): the percentage of diaphragm thickness change during inspiration and expiration, measured by bedside ultrasound at the seventh to eighth intercostal spaces on the right midaxillary line [29]; (4) ICU length of stay (ILOS): the total time from ICU admission to discharge; (5) rapid shallow breathing index (RSBI): the ratio of respiratory rate to tidal volume, measured during spontaneous breathing trial [30]; (6) tracheostomy rate: the proportion of patients who underwent tracheostomy during hospitalization; (7) adverse events: including life-threatening adverse events, procedure-related adverse events and minor adverse events, recorded according to the definition in each included study.

### 2.5. Risk-of-Bias Assessment

The methodological quality and risk of bias of the included studies were independently assessed by two reviewers using the Cochrane Risk of Bias tool version 2 (RoB 2) [31]. This tool assesses five key domains: (1) the randomization process; (2) deviations from intended interventions; (3) missing outcome data; (4) outcome measurement; (5) selection of the reported result. For each domain, a judgment of “low risk of bias”, “some concerns”, or “high risk of bias” was assigned, guided by a series of signaling questions. An overall risk-of-bias rating for each study was then derived from these domain-specific assessments. Disagreements between the two reviewers were resolved through discussion or through consultation with a third reviewer to reach a consensus. The risk-of-bias assessments were incorporated into the interpretation of the review findings. Additionally, sensitivity analyses were carried out to evaluate the robustness of the findings in relation to study quality.

### 2.6. Certainty of Evidence Assessment

Two independent reviewers systematically assessed the certainty of evidence for all outcomes using the Grading of Recommendations Assessment, Development, and Evaluation (GRADE) framework [32], with disagreements resolved by a third reviewer. This framework included a comprehensive assessment of five key domains: risk of bias, inconsistency, indirectness, imprecision, and publication bias. Based on these evaluations, the quality of the evidence was graded into four levels (high, moderate, low, or very low) using the GRADEpro tool. Finally, an evidence profile was constructed in accordance with the Cochrane Handbook.

### 2.7. Statistical Analysis

Meta-analysis was performed using Review Manager (RevMan 5.4, Copenhagen, Denmark: The Nordic Cochrane Centre, The Cochrane Collaboration 2014). Although newer web-based versions were available, RevMan 5.4 was selected because it was an offline tool that supported the statistical methods required for this analysis. Pooled odds ratios (ORs) with 95% confidence intervals (95% CIs) were calculated for dichotomous outcomes, whereas mean differences (MDs) with 95% CIs were used for continuous outcomes. When necessary, medians and interquartile ranges were converted to estimated means and standard deviations using established formulas [33]. Heterogeneity was assessed using the Chi-square test and quantified with the *I*^2^ statistic. An *I*^2^ value greater than 50% or a Chi-square *p*-value less than 0.10 was considered indicative of considerable heterogeneity, in which case a random-effects model (REM) was applied; otherwise, a fixed-effects model (FEM) was used [34]. For outcomes where data were insufficient for meta-analysis or quantitative pooling was inappropriate, a narrative synthesis was performed. Forest plots were generated for each outcome to summarize effect sizes and confidence intervals, and subgroup analyses were conducted to explore potential sources of heterogeneity. Sensitivity analyses were performed to examine the robustness of the pooled results. Assessment of publication bias using funnel plots, Egger’s test, and Begg’s test was planned when the number of included studies exceeded 10 [35].

## 3. Results

### 3.1. Literature Identification and Selection

**Figure 1:**
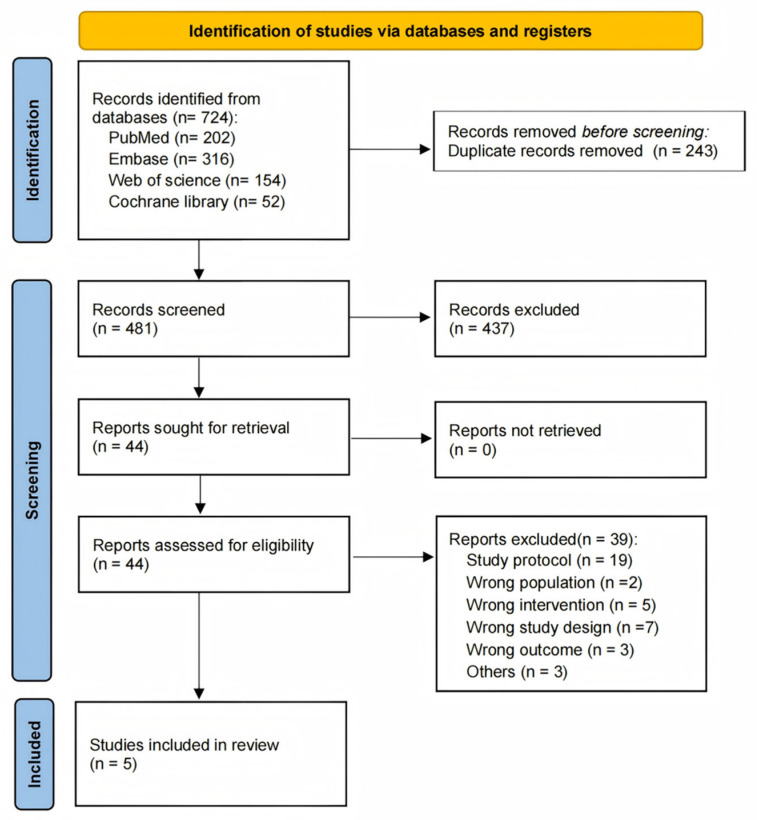
PRISMA flow chart.

The initial literature search identified a total of 724 citations. Following duplicate removal, 481 citations were screened by title and abstract. Subsequently, 437 articles were excluded based on eligibility criteria. Next, the full texts of the remaining 44 articles were assessed. Following the full-text review, 39 articles were excluded. To ensure strict adherence to our PICOS criteria, the specific reasons for exclusion were detailed as follows: wrong population (*n* = 2, involving animal models or healthy volunteers); wrong intervention (*n* = 5, utilizing non-targeted peripheral or abdominal neuromuscular electrical stimulation rather than diaphragm-targeted PNS); study protocols without published results (*n* = 19); wrong study designs such as non-randomized observational trials (*n* = 7); wrong outcomes lacking predefined clinical endpoints (*n* = 3); and other reasons (*n* = 3). Ultimately, five studies met all inclusion criteria and were included in the meta-analysis (Figure 1).

### 3.2. Study Characteristics

A total of five randomized controlled trials (RCTs) published between 2022 and 2026 were included [17,18,19,20,21]. Among them, three were single-center RCTs [17,18,19], and two were multicenter RCTs [20,21]. The studies originated from China (2 studies [17,18]), France (1 study [19]), Brazil (1 study [20]), and the United States/Europe (1 study [21]). A total of 431 participants were included, comprising 211 in the intervention group and 220 in the control group, with individual study sample sizes ranging from 44 to 216. The mean or median age of participants ranged between 60 and 80 years across all studies. The required duration of mechanical ventilation at enrollment varied substantially. Specifically, two studies included patients who had received ≥ 24 h of mechanical ventilation [19,20], one study required ≥ 3 days [18], one study included patients with ≥96 h and difficult weaning [21], and one study specifically enrolled patients with prolonged mechanical ventilation (>21 days) [17]. Interventions (PNS) included TEDS (3 studies [17,19,20]), EDP (1 study [18]), and TTDN (1 study [21]). Control groups received either sham stimulation (2 studies [17,19]) or standard of care (3 studies [18,20,21]). Stimulation parameters significantly differed across studies: frequency ranged from 15 Hz to 50 Hz, and pulse width ranged from 200 μs to 400 μs. Intensity was generally titrated based on patient tolerance or visible diaphragmatic contraction. Treatment protocols varied, typically consisting of 1–2 daily sessions lasting 10 to 30 min. Regarding outcome reporting, all five studies reported successful weaning rates and adverse events. Four studies reported MIP [17,19,20,21] and three studies [18,19,20] reported DTF. Four studies reported ICU length of stay [17,18,19,21]. Two studies reported the RSBI and tracheostomy rate. Detailed characteristics of the included studies are listed in Table 2.

### 3.3. Risk-of-Bias Assessment Results

**Figure 2:**
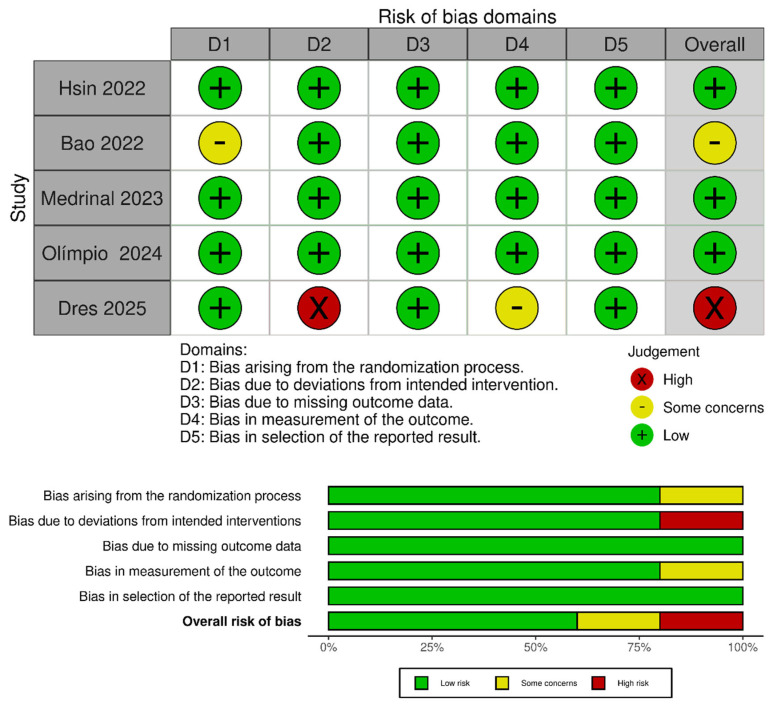
Risk-of-bias assessment table and graph of the included studies.

The methodological quality of the five included RCTs was independently assessed using the Cochrane RoB 2 (Risk of Bias 2) tool, with the results visually summarized in Figure 2. Overall, the methodological quality of the included studies was heterogeneous. Three studies were judged to have an overall “low risk of bias” [17,19,20]. At the same time, one study was rated as having “some concerns”, primarily due to insufficient detail regarding the allocation concealment process [18]. One study was judged to be at “high risk of bias”, largely due to its “open-label” design, which introduced a “high risk of bias” in the domain of deviation from intended interventions and “some concerns” in the domain of outcome measurement [21]. In the domains of missing outcome data and selective reporting, all five studies were rated as “low risk” (Figure 2).

### 3.4. Outcome

#### 3.4.1. Weaning Success Rate

**Figure 3:**
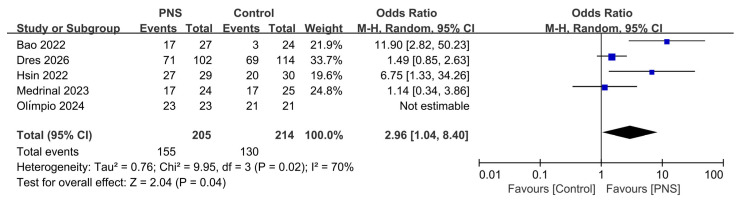
Forest plot comparing the weaning success rate between the PNS and control groups. PNS = phrenic nerve stimulation; M-H = Mantel-Haenszel method; CI = confidence interval.

The weaning success rate was compared between the PNS and control groups across five studies [17,18,19,20,21]. Considerable heterogeneity was observed (*χ*^2^ = 9.95, *p* = 0.02, *I*^2^ = 70%). The random-effects model showed that PNS was associated with a higher weaning success rate than the control group [*OR* = 2.96 (1.04, 8.40), *Z* = 2.04, *p* = 0.04] (Figure 3).

To explore potential sources of heterogeneity, subgroup analysis was conducted. When stratified by the invasiveness of the intervention, the noninvasive subgroup showed a point estimate favoring PNS, but the difference was not statistically significant [*OR* = 4.27 (0.96, 19.06), *Z* = 1.90, *p* = 0.06], with considerable heterogeneity (*χ*^2^ = 6.65, *p* = 0.04, *I*^2^ = 70%). The invasive subgroup showed no statistically significant difference compared with the control group [*OR* = 1.49 (0.85, 2.63), *Z* = 1.39, *p* = 0.16]. The test for subgroup differences was not statistically significant (*χ*^2^ = 1.66, *p* = 0.20) (Supplementary Figure S1).

Furthermore, when stratified by the baseline duration of mechanical ventilation, the prolonged mechanical ventilation subgroup showed higher odds of weaning success with PNS than with control [*OR* = 4.32 (1.01, 18.52), *Z* = 1.97, *p* = 0.05], with considerable heterogeneity (*χ^2^* = 8.98, *p* = 0.01, *I^2^* = 78%). In the MV ≥ 24 h subgroup, no statistically significant difference was observed between the two groups [*OR* = 1.14 (0.34, 3.86), *Z* = 0.22, *p* = 0.83]. The test for subgroup differences was not statistically significant (*χ^2^* = 1.89, *p* = 0.17) (Supplementary Figure S2).

To assess the robustness of these findings, a sensitivity analysis was conducted by excluding the study with the largest sample size (Dres et al.). Heterogeneity remained considerable (*χ*^2^ = 6.65, *p* = 0.04, *I*^2^ = 70%), and under the random-effects model, the pooled effect was not statistically significant [*OR* = 4.27 (0.96, 19.06), *Z* = 1.90, *p* = 0.06]. Based on the GRADE framework, the overall certainty of evidence for the weaning success rate was evaluated as low (Supplementary Table S3).

#### 3.4.2. Duration of Mechanical Ventilation (DMV)

**Figure 4:**
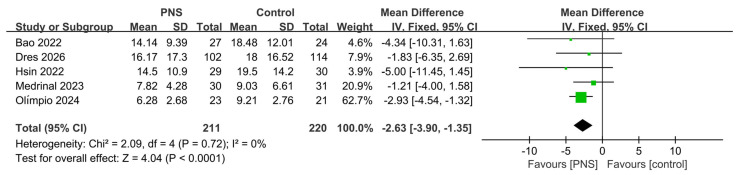
Forest plot for the duration of mechanical ventilation between the PNS and control groups. PNS = phrenic nerve stimulation; IV = inverse variance method; CI = confidence interval; SD = standard deviation.

Mechanical ventilation duration between the two groups was examined across five studies [17,18,19,20,21]. No statistical heterogeneity was detected (*χ*^2^ = 2.09, *p* = 0.72, *I*^2^ = 0%). The fixed-effects model showed a shorter duration of mechanical ventilation in the PNS group compared with the control group [*MD* = −2.63 (−3.90, −1.35), *Z* = 4.04, *p* < 0.001] (Figure 4). As a sensitivity analysis, a random-effects model was applied to account for potential clinical heterogeneity, which showed similar results [*MD* = −2.63 (−3.90, −1.35), *p* < 0.001] (Supplementary Figure S3). According to the GRADE framework, the certainty of evidence for the duration of mechanical ventilation was assessed as high (Supplementary Table S3).

#### 3.4.3. Maximal Inspiratory Pressure (MIP)

**Figure 5:**
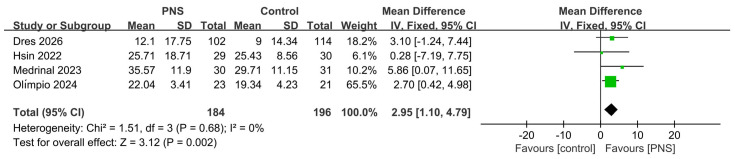
Forest plot for maximal inspiratory pressure between the PNS and control groups. PNS = phrenic nerve stimulation; IV = inverse variance method; CI = confidence interval; SD = standard deviation.

Moreover, MIP was compared between the two groups across four studies [17,19,20,21]. Likewise, no statistical heterogeneity was observed (*χ*^2^ = 1.51, *p* = 0.68, *I*^2^ = 0%). Under the fixed-effects model, the PNS group had a higher MIP than the control group [*MD* = 2.95 (1.10, 4.79), *Z* = 3.12, *p* = 0.002] (Figure 5). A sensitivity analysis using a random-effects model showed similar results [*MD* = 2.95 (1.10, 4.79), *p* = 0.002] (Supplementary Figure S4). The certainty of evidence for maximal inspiratory pressure was rated as moderate based on the GRADE assessment (Supplementary Table S3).

#### 3.4.4. Diaphragm Thickening Fraction (DTF)

DTF was compared between the two groups across three studies [18,19,20]. Considerable heterogeneity was observed (*χ*^2^ = 5.27, *p* = 0.07, *I*^2^ = 62%), and under the random-effects model, the PNS group had higher DTF values than the control group [*MD* = 15.67 (4.84, 26.50), *Z* = 2.84, *p* = 0.005].

**Figure 6:**
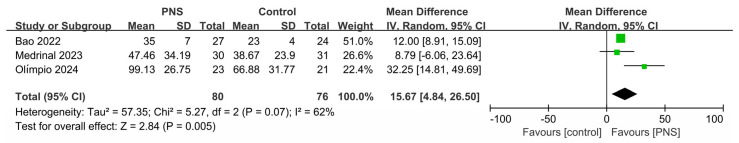
Forest plot for the diaphragm thickening fraction between the PNS and control groups. PNS = phrenic nerve stimulation; IV = inverse variance method; CI = confidence interval; SD = standard deviation.

To assess the robustness of these findings, a sensitivity analysis was conducted by excluding the study with the largest sample size (Medrinal et al.). The heterogeneity remained considerable (*χ*^2^ = 5.02, *p* = 0.03, *I*^2^ = 80%), and under the random-effects model, the pooled effect remained statistically significant [*MD* = 20.23 (0.74, 39.73), *Z* = 2.03, *p* = 0.04]. Another sensitivity analysis was conducted by excluding Olímpio’s study, which reduced heterogeneity (*χ*^2^ = 0.17, *p* = 0.68, *I*^2^ = 0%) while maintaining a statistically significant pooled effect [*MD* = 11.87 (8.84, 14.89), *Z* = 7.69, *p* < 0.001]. The excluded Olímpio’s study used a 60 min daily stimulation (2 sessions × 30 min), which was three times the duration of the 20 min daily protocol used in the studies by Bao et al. (2 sessions × 10 min) and Medrinal et al. (1 session × 20 min) (Figure 6). Based on the GRADE framework, the certainty of evidence for the diaphragm thickening fraction was evaluated as low (Supplementary Table S3).

#### 3.4.5. ICU Length of Stay (ILOS)

**Figure 7:**
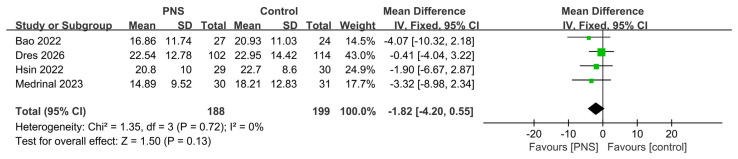
Forest plot for the ICU length of stay between the PNS and control groups. PNS = phrenic nerve stimulation; IV = inverse variance method; CI = confidence interval; SD = standard deviation.

ICU length of stay was compared between the two groups across four studies [17,18,19,21]. No statistical heterogeneity was observed (*χ*^2^ = 1.35, *p* = 0.72, *I*^2^ = 0%), and under the fixed-effects model, no statistically significant difference was observed in ICU length of stay between the PNS group and the control group [*MD* = −1.82 (−4.20, 0.55), *Z* = 1.50, *p* = 0.13] (Figure 7). When reanalyzed using a random-effects model as a sensitivity analysis, the difference between the two groups remained not statistically significant [*MD* = −1.82 (−4.20, 0.55), *p* = 0.13] (Supplementary Figure S5). According to the GRADE framework, the certainty of evidence for ICU length of stay was assessed as moderate (Supplementary Table S3).

#### 3.4.6. Rapid Shallow Breathing Index (RSBI)

**Figure 8:**
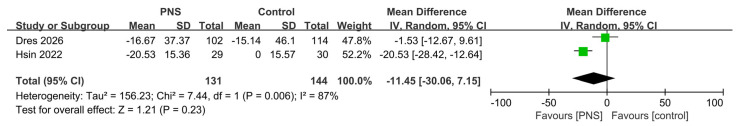
Forest plot for the rapid shallow breathing index between the PNS and control groups. PNS = phrenic nerve stimulation; IV = inverse variance method; CI = confidence interval; SD = standard deviation.

RSBI was compared between the two groups across two studies [17,21]. Considerable heterogeneity was observed (*χ*^2^ = 7.44, *p* = 0.006, *I*^2^ = 87%), and under the random-effects model, no statistically significant difference was observed between the two groups [*MD* = −11.45 (−30.06, 7.15), *Z* = 1.21, *p* = 0.23]. Given that only two studies were included for this outcome, it was not feasible to determine the source of heterogeneity through sensitivity or subgroup analyses (Figure 8). The certainty of evidence for the rapid shallow breathing index was rated as low based on the GRADE assessment (Supplementary Table S3).

#### 3.4.7. Tracheostomy Rate

**Figure 9:**
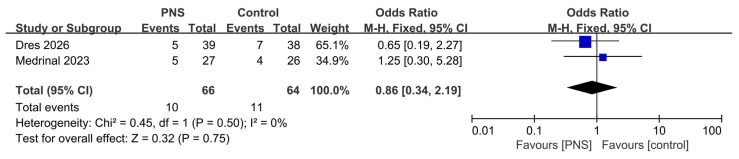
Forest plot for the tracheostomy rate between the PNS and control groups. PNS = phrenic nerve stimulation; M-H = Mantel-Haenszel method; CI = confidence interval.

Furthermore, the tracheostomy rate was compared between the two groups across two studies [19,21]. No statistical heterogeneity was observed (*χ*^2^ = 0.45, *p* = 0.50, *I*^2^ = 0%). The fixed-effects model showed no statistically significant difference in tracheostomy rate between the PNS and control groups [*OR* = 0.86 (0.34, 2.19), *Z* = 0.32, *p* = 0.75] (Figure 9). A sensitivity analysis applying a random-effects model also showed no statistically significant difference [*OR* = 0.86 (0.34, 2.21), *p* = 0.76] (Supplementary Figure S6). Based on the GRADE framework, the certainty of evidence for the tracheostomy rate was evaluated as low (Supplementary Table S3).

#### 3.4.8. Adverse Events

Adverse events were reported in all five included RCTs. The safety profiles differed depending on the device type (noninvasive versus invasive modalities). Regarding noninvasive transcutaneous stimulation (TEDS/EDP), four RCTs reported that the intervention was tolerated. Three studies reported a 0% incidence of device-related adverse events. One study reported slight, non-harmful interference with cardiac monitoring signals. No procedure-related or life-threatening adverse events were recorded in any of the noninvasive trials. In contrast, regarding the invasive transvenous modality, the single RCT evaluating reported TTDN a higher incidence of serious adverse events in the treatment group compared with the control group (35.8% vs. 23.7%). Notably, serious procedure-related harms were documented, including potentially fatal events such as hemothorax, pneumohemothorax, and tension pneumothorax, leading to acute coronary syndrome, which were reported to be associated with the central venous catheter placement required for the device.

## 4. Discussion

This study synthesized data from five RCTs involving 431 patients and evaluated the efficacy and safety of PNS in mechanically ventilated patients. The results suggested that PNS was associated with a higher weaning success rate, higher MIP and DTF values, and a shorter duration of mechanical ventilation. However, PNS was not associated with statistically significant differences in ICU length of stay, RSBI, or tracheostomy rate. Regarding safety, noninvasive stimulation was generally tolerated in the included studies, whereas invasive transvenous stimulation was associated with procedure-related serious adverse events in the included trial.

PNS may improve the weaning success rate in MV patients, with its core mechanism attributed to the active mitigation of VIDD. PNS induces rhythmic contraction of the diaphragm through neuromodulation, simulating physiological respiratory movements [11,36]. Disuse atrophy in mechanically ventilated patients can develop within the first 18 to 24 h of inactivity. This process involves the upregulation of proteolytic systems (such as the ubiquitin-proteasome pathway), oxidative stress, and mitochondrial dysfunction. Although conventional lung-protective ventilation provides pulmonary safety, it may not adequately prevent this muscular degradation. By promoting active contractions, PNS may mitigate this process. Current translational and clinical evidence indicates that PNS may not completely prevent muscle wasting, particularly in the presence of systemic inflammation or sepsis, but may attenuate disuse atrophy [37]. It may reduce muscle fiber degradation, limit the pathological shift in muscle fiber types, and help preserve muscle mass. These effects were consistent with the higher DTF values observed in our meta-analysis [38]. Therefore, PNS may represent a potential strategy for attenuating diaphragmatic atrophy, although it may not completely prevent muscle loss in patients with complex critical illness.

The impact of this mechanism on clinical outcomes was supported by the relevant studies: Hsin et al. enrolled PMV patients with ≥ 21 days of mechanical ventilation and showed a higher weaning success rate in the TEDS group compared with the control group [17]. Likewise, Wu et al. reported a higher weaning success rate in the PNS group than in the control group [39]. Taken together, these findings suggested that PNS was associated with improved weaning outcomes, possibly through effects on diaphragmatic strength and structure [40]. Nevertheless, considerable heterogeneity was observed among the included studies. Subgroup analysis based on invasiveness suggested a more favorable trend for noninvasive modalities (TEDS/EDP) compared to the invasive modality (TTDN). However, the subgroup difference was not statistically significant, so this finding should be interpreted cautiously. The lack of observed benefit in the invasive TTDN study, which carried the highest weight, may have been related to the complexity of its difficult-to-wean population. In these patients, weaning failure may have been influenced by ICU-acquired weakness (ICU-AW), neuropathy, systemic illness, or other factors, rather than isolated VIDD [41]. Consequently, although PNS may have increased diaphragm strength, this effect may not have been sufficient to improve weaning outcomes in this specific group. Furthermore, subgroup analysis based on the baseline duration of mechanical ventilation indicated that patients with prolonged ventilation might derive greater benefit from PNS than those with MV ≥ 24 h. However, the test for subgroup differences was not statistically significant. In prolonged ventilation, VIDD may have contributed to weaning difficulty and may have represented a partially reversible target for PNS. In contrast, in patients at earlier ventilation stages, weaning outcomes may have been influenced by unresolved acute disease, systemic inflammation, or other non-diaphragmatic factors [42]. Additionally, the lack of positive results reported in early ventilation studies, such as that by Medrinal et al., may have been related to an insufficient intervention duration. Sensitivity analysis indicated that the pooled results were not fully robust and may have been influenced by individual studies. These findings suggested that future studies need to define target populations by intervention modality, ventilation duration, and the relative contribution of VIDD to weaning failure.

Our analysis showed a statistically significant difference in MIP following PNS; however, the magnitude of this change was small, and its clinical relevance remained uncertain [43]. MIP is an important indicator of global inspiratory muscle strength, and PNS may affect inspiratory muscle function by inducing passive diaphragm contractions and mitigating diaphragmatic disuse atrophy associated with VIDD [44]. Nevertheless, this finding should be interpreted cautiously. The pooled mean difference in MIP was only 2.95 cmH_2_O, and a universally accepted minimal clinically important difference for MIP in mechanically ventilated ICU patients had not been established. Given the effort-dependent nature of MIP measurement and potential measurement variability, this small statistically significant difference may not translate into clinically meaningful benefit or improved weaning outcomes. Future large-scale trials are needed to assess whether changes in MIP after PNS exceed measurement variability and whether they are associated with patient-centered clinical outcomes.

DTF is a commonly used ultrasonographic indicator for assessing diaphragm function. It reflects the degree of diaphragm thickening during contraction [45]. PNS may help maintain diaphragm thickness and influence contraction efficiency through regular neural stimulation. This interpretation was partly consistent with findings from relevant studies. Shen et al. [46] found that the DTF in the PNS group was significantly higher than in the control group after 5 days of intervention [(25.34 ± 2.38)% vs. (23.93 ± 2.33)%]. Similarly, Virolle et al. [47] reported that TFdi, which corresponds to DTF, was higher in the complete weaning group at the last measurement [23 (19–46)%] than in the partial weaning and death group [16 (14–22)%]. These findings suggested that higher DTF values may be associated with better diaphragm function and weaning outcomes, but they did not establish a causal relationship. However, in our meta-analysis, the pooled evaluation of DTF should be considered exploratory. The corresponding conclusion was limited by the inclusion of only three eligible studies and considerable statistical heterogeneity. This heterogeneity may have been related to methodological differences, particularly stimulation dose and ultrasound assessment methods. First, regarding stimulation dose and protocol, the larger effect size observed in the study by Olímpio et al. [20], compared with those by Bao et al. [18] and Medrinal et al. [19], may have been associated with the longer intervention duration used in that study. Olímpio et al. [20] used 60 min/day, whereas the other two studies used shorter durations of 15–30 min/day. Sensitivity analysis excluding the study by Olímpio et al. showed that heterogeneity decreased to 0%. These findings suggested that the association between PNS and DTF may be partly related to intervention duration. Second, differences in ultrasound methods may have limited comparability across studies. Variations in ultrasound transducer frequency across trials may have affected measurement consistency. In addition, identifying the zone of apposition and capturing the same respiratory phase were operator-dependent. These factors may have introduced measurement variability. Therefore, the DTF findings should be interpreted cautiously and require confirmation in studies with standardized ultrasound protocols.

Notably, PNS may shorten the duration of mechanical ventilation, with a high degree of homogeneity among the studies. By affecting diaphragmatic function, PNS may reduce patient dependence on MV, thereby accelerating the weaning process [48]. This may stem from an improvement in diaphragm contractile efficiency, which can partly alleviate respiratory muscle fatigue [12]. This conclusion was also supported by relevant studies: Olímpio et al. noted that the MV duration in the TEDS group was 2.93 days shorter than in the control group [20]; Dres et al. similarly observed that the ventilation duration was 2.5 days shorter in the PNS group than in the control group [21]. The low heterogeneity suggests that all studies, regardless of stimulation method, show a similar trend, thus positioning PNS as a potentially useful intervention to reduce the burden of MV.

No significant difference was noted in ICU length of stay between the PNS and control groups, which may be attributed to ICU length of stay being influenced by numerous factors. In addition to the duration of mechanical ventilation, the severity of underlying conditions (e.g., sepsis, ARDS), the incidence of complications (e.g., VAP, cardiovascular events), and discharge preparation processes may all offset the potential shortening effect of PNS [49,50,51]. For example, Dres et al. demonstrated that although the ventilation duration in the PNS group was shortened [21], its intervention-related serious complications (e.g., catheter placement risks) might also lead to an extension of ICU length of stay, thereby offsetting the potential benefits associated with shorter MV duration. This highlights the need to balance the benefits and risks of PNS in the clinical setting. As an indicator of weaning outcomes, no significant difference was observed in the RSBI between the two groups, accompanied by considerable heterogeneity. Only two studies were included in this analysis, and the inconsistency may result from inconsistent RSBI measurement methods (e.g., measurements under different ventilation modes or variations in measurement time points) or differences in patients’ respiratory drive [52,53]. Of note, no significant difference was observed in tracheostomy rate between the two groups. This may be ascribed to the primary indications for tracheostomy, including the need for airway protection (e.g., coma, neurological injury) or airway stenosis, which are not directly related to diaphragm function [54]. PNS may improve diaphragmatic function and cannot modify airway structure or neurological status; therefore, no change in tracheostomy rate would be expected.

The safety profile of PNS may vary depending on the invasiveness of the device utilized. To mitigate the risk of ventilator-induced diaphragm dysfunction in clinical practice, noninvasive phrenic nerve stimulation modalities may offer a feasible approach. Our analysis distinguishes the safety profile of noninvasive modalities from the procedural risks associated with invasive transvenous stimulation. Unlike invasive approaches, noninvasive techniques use surface electrodes applied directly to the neck or thorax. This may facilitate earlier implementation during the mechanical ventilation course, allowing clinicians to intervene in the early stages of disuse atrophy. In the reviewed studies, noninvasive stimulation was associated with reported tolerability and a low incidence of device-related adverse events. These findings suggested that noninvasive modalities may warrant further evaluation in intensive care rehabilitation settings. In contrast, invasive PNS was associated with procedural risks. These findings indicate that invasive PNS may have important procedure-related harms, and its use should be carefully weighed against its potential physiological benefits. The requirement for central venous catheterization may introduce potential complications, such as vascular injury, tension pneumothorax, and catheter-related bloodstream infections (CRBSI) [21]. The potential physiological benefits of invasive modalities should be interpreted in relation to these procedural risks. Given the available safety data, noninvasive modalities may be prioritized in future studies or selected clinical contexts. Invasive methods may require careful patient selection and procedural risk mitigation. Assisted visualization techniques, such as real-time ultrasound guidance, may help reduce catheter-related complications [55].

The findings of this study are consistent with the conclusions of the meta-analysis by Tong et al. [25]. Indeed, both studies suggested that PNS was associated with a higher weaning success rate in mechanically ventilated patients, a shorter duration of mechanical ventilation, and higher MIP. These findings collectively support the potential role of PNS in promoting mechanical ventilation weaning by countering diaphragmatic disuse atrophy and enhancing respiratory muscle strength [56]. However, this study has advantages in methodological rigor and analytical depth. First, Tong et al. only qualitatively described MIP and did not evaluate other indicators of diaphragm function. In contrast, this study quantitatively pooled physiological indicators, including MIP and DTF. The results indicated that PNS was associated with higher MIP and DTF values, providing additional information on its potential physiological effects from functional and structural perspectives. Second, Tong et al. included both RCTs and cohort studies, which may have increased clinical and methodological heterogeneity. Their reported heterogeneity was high for primary outcomes, including DMV (*I^2^* = 99%) and ILOS (*I^2^* = 98%). This may have affected the certainty of the pooled estimates. In comparison, this study included only RCTs and showed lower heterogeneity for DMV (*I*^2^ = 0%) and ILOS (*I*^2^ = 0%). In summary, this study provided a more focused synthesis based on RCT evidence and included additional physiological outcomes related to diaphragm function.

This meta-analysis has several strengths. First, it features comprehensive outcome inclusion, covering efficacy (weaning success rate, ventilation duration, diaphragm function) and safety (adverse events), alongside clinical outcomes such as ICU length of stay and tracheostomy rate. Additionally, it included assessments of DTF and RSBI, which were not fully evaluated in some previous reviews. Second, the analysis explored clinical and statistical heterogeneity. For instance, subgroup analyses examined intervention invasiveness and baseline ventilation duration as potential sources of heterogeneity for the weaning success rate, while sensitivity analysis revealed that intervention duration was the primary contributor to heterogeneity in the DTF outcome. Third, the RCT-only design improved methodological consistency for several pooled estimates; by including only RCTs, key outcomes such as mechanical ventilation duration and MIP showed low statistical heterogeneity, which supported the consistency of these pooled estimates. Finally, we employed the updated Cochrane Risk of Bias 2 tool and the GRADE framework to assess the methodological quality and the certainty of evidence for each outcome, which improved the transparency of the evidence assessment.

However, this study also has several limitations. First, the limited number of included studies resulted in small, pooled sample sizes for secondary outcomes such as the rapid shallow breathing index and the tracheostomy rate. Additionally, the primary outcome of weaning success appeared to be influenced by the inclusion of a single large trial, indicating that the current evidence should be interpreted cautiously. Moreover, small-study effects and publication bias could not be formally assessed because of the limited number of included studies. Small trials with favorable or statistically significant findings may also have been more likely to be published. Second, methodological and interventional variability was present across the primary studies. Differences in stimulation modes, treatment doses, and safety assessment criteria limited the comparability of intervention protocols. Furthermore, inherent study design limitations may introduce reporting and selection biases. Specifically, the impossibility of blinding in the invasive trial and a non-transparent randomization process in another study compromise the overall reliability of the findings. Third, because our meta-analysis relied on aggregate study-level data rather than individual patient records, we were unable to adjust for patient-level factors that may influence weaning success. These factors include illness severity, APACHE II and SOFA scores, depth and duration of sedation, sepsis, ARDS, neuromuscular blocker use, ICU-acquired weakness, baseline diaphragm dysfunction, and nutritional status, which were either unavailable or inconsistently reported across the included trials. Finally, the current literature mainly reports immediate physiological indicators and short-term weaning-related outcomes, while sustained clinical outcomes remain inadequately reported. Critical endpoints, including overall mortality, reintubation after 48 h, ICU readmission, functional recovery following discharge, and quality of life, were not consistently assessed across the included trials, preventing a comprehensive evaluation of the long-term therapeutic benefits of PNS.

Advanced respiratory support represents a potential area for future PNS research. Mechanical ventilation remains a commonly used life-support modality, and ECMO may provide rescue support in selected patients with severe respiratory failure. However, prolonged respiratory support can reduce physiological diaphragm activity and contribute to ventilator-induced diaphragmatic dysfunction [57]. Because ECMO patients and pediatric ICU populations were not evaluated in the included trials, these applications should be interpreted as future research areas rather than conclusions supported by the current meta-analysis. Future studies could evaluate whether PNS can be integrated with advanced ventilation strategies, such as neurally adjusted ventilatory assist and proportional assist ventilation [58]. In such synchronized systems, trigger lag may need to be assessed. This may require monitoring the delay between neural inspiratory effort, ventilator pressurization, and phrenic stimulation. Potential tools include ventilator pressure-flow waveforms, esophageal pressure monitoring, electrical activity of the diaphragm, and electrical impedance tomography (EIT) [59].

Based on the identified limitations, future research could focus on four key areas. First, subsequent multicenter trials should consider improving research methodology by adopting blinded designs and standardizing core intervention variables, including stimulation parameters and treatment durations. Second, targeted investigations are needed to identify patient populations that may benefit from PNS, such as those requiring prolonged mechanical ventilation or those with underlying chronic respiratory conditions. These studies should also continue to monitor the procedural safety of invasive modalities in a standardized manner. Third, future study designs should include longer-term patient-centered endpoints, such as survival, functional recovery, and quality of life after discharge. Finally, the clinical application of this technology may require further evaluation of combined and synchronized intervention strategies. Studies could assess whether neural stimulation combined with early mobilization or inspiratory muscle training is associated with improved respiratory rehabilitation outcomes. In addition, integrating stimulation with the electrical activity of the diaphragm may be explored as a way to provide proportional assistance and reduce patient–ventilator asynchrony.

In summary, this meta-analysis showed that PNS was associated with a higher weaning success rate, shorter mechanical ventilation duration, and higher MIP and DTF values in mechanically ventilated patients. However, no statistically significant differences were observed in ICU length of stay, RSBI, or tracheostomy rate. Noninvasive stimulation was generally tolerated in the included studies, whereas transvenous stimulation was associated with procedure-related risks. The current evidence remains limited by the small number of studies and clinical heterogeneity. Therefore, routine clinical use of PNS should await confirmation by larger, blinded, multicenter RCTs with standardized protocols that assess its long-term efficacy and safety.

## Figures and Tables

**Table 1:** Eligibility criteria based on the PICOS framework.

PICOS	Inclusion Criteria	Exclusion Criteria
Population (P)	Adult patients (≥18 years) admitted to the ICU who required mechanical ventilation.	Patients under 18 years of age or those not requiring mechanical ventilation.
Intervention (I)	Phrenic nerve stimulation (PNS), including transcutaneous approaches (e.g., TEDS, EDP) and transvenous approaches (e.g., TTDN).	Interventions other than phrenic nerve stimulation (e.g., pacemakers, or other forms of neuromuscular electrical stimulation).
Comparison (C)	Standard of care or sham stimulation.	No control group or inappropriate control group.
Outcomes (O)	Primary: Weaning success rate. Secondary: Duration of mechanical ventilation (DMV), maximal inspiratory pressure (MIP), diaphragm thickening fraction (DTF), ICU length of stay (ILOS), rapid shallow breathing index (RSBI), tracheostomy rate, and adverse events.	Lack of reported data on the predefined outcomes.
Study Design (S)	Randomized controlled trials (RCTs).	Non-randomized controlled trials (e.g., case reports, observational studies, or reviews); conference abstracts.

**Table 2:** Characteristics of included trials.

Author (Year)	Region	Study Design	N/Male	Sample Size (PNS/Control)	Age (PNS/Control)	MV Duration	PNS	Control	PNS Protocol				Outcome
									Device	Settings	Stimulation Site	Duration	
Hsin 2022 [17]	China	Single-center RCT	59/43	29/30	73.3 ± 12.3/77.1 ± 8.2	>21 days	TEDS	Sham stimulation	Commercial stimulator (Omnistim 500, ZMI, Taiwan) applying biphasic waves	Frequency: 30 Hz; Pulse width: 400 μs; Rise time: 0.7 s; Intensity: Gradually increased until visible muscle contraction	1. Parasternal region beside the xiphoid process; 2. The sixth and seventh intercostal spaces in line with the mid-axillary line	Daily: 1 session/day, 30 min/session; Weekly: 5 days/week; Total: Until the end of the weaning trial	①②③④⑥⑦
Bao 2022 [18]	China	Single-center RCT	51/21	27/24	60.04 ± 13.47/66.33 ± 14.45	≥3 days	EDP	Standard of care	EDP device (Arahelio Biotechnology Developing Guangzhou Co., Ltd., Guangzhou, China)	Frequency: 40 Hz/30 min/time; Intensity: From low to high, adjusted to maximum diaphragm movement via ultrasound	1. Pacing electrode pasted on the outer third of the lower end of the sternocleidomastoid muscle; 2. Auxiliary electrode placed in the second intercostal space of the midclavicular line	Daily: 2 times/day, 10 min/time; Weekly: 5 days/week; Total: Until successful weaning, death, transfer out of ICU, or 28 days	①②③⑤⑥
Medrinal 2023 [19]	France	Single-center RCT	61/46	30/31	62 ± 10/61 ± 15	≥24 h	TEDS	Sham stimulation	Device providing bidirectional current	Frequency: 50 Hz; Pulse width: 300 μs; Stimulation cycle: 6 s stimulation + 10 s rest	Two pairs of 5 × 5 cm electrodes applied to each hemithorax. First pair: above and below the xiphoid process (8th–10th anterior intercostal spaces). Second pair: over the medio-axillary line (8th–10th intercostal spaces)	Daily: 1 session/day, 20 min/session; Weekly: 5 days/week; Total: Until the first extubation attempt	①②③④⑤⑥⑧
Olímpio 2024 [20]	Brazil	Multicenter RCT	44/16	23/21	69 (60–86)/66 (60–79)	≥24 h	TEDS	Standard of care	Phrenics equipment (Dualpex 961, Quark^®^, São Paulo, Brazil)	Frequency: 30 Hz; Pulse width: 0.4 ms; Respiratory rate: 15 bpm; Time parameters: Hold time 1 s, rise time 1 s, fall time 2 s, no-stimulation time 2 s	1. Two electrodes placed in the right and left paraxiphoid region; 2. Another two electrodes placed in the direction of the axillary midline over the seventh intercostal space (right and left sides)	Daily: 2 times/day (6 h interval between sessions), 30 min/time; Total: Until extubation	①②③④⑤
Dres 2026 [21]	US, Europe	Multicenter RCT	216/145	102/114	64.6 ± 12.1/63.8 ± 11.5	≥96 h	TTDN	Standard of care	1. TTDN device (Lungpacer Medical Inc., Vancouver, BC, Canada); 2. Sterile multi-electrode central venous catheter (Keystone/AeroPace System)	Frequency: 15 Hz; Pulse width: 200–300 μs; Intensity: ≤27 mA (max tolerated by patient); Stimulation pattern: 6 sets × 10 stimulations/session	Bilateral phrenic nerve stimulation: 1. Catheter inserted via the left subclavian or left jugular vein; 2. Tip positioned just above the cavoatrial junction	Daily: 2 sessions/day (total 120 stimulations/day); Total: Up to 30 days or until successful weaning, death, or withdrawal	①②③④⑤⑥⑦⑧

Note: RCT = Randomized controlled trial; DMV = Duration of mechanical ventilation; TEDS = Transcutaneous electrical diaphragmatic stimulation; TTDN = Temporary transvenous diaphragm neurostimulation; EDP = External diaphragmatic pacing. ① Weaning success rate; ② Adverse events; ③ DMV; ④ MIP = Maximal inspiratory pressure; ⑤ DTF = Diaphragm thickening fraction; ⑥ ILOS = ICU length of stay; ⑦ RSBI = Rapid shallow breathing index; ⑧ Tracheostomy rate.

## Data Availability

No new data were generated or analyzed in this study. Therefore, data sharing is not applicable to this article.

## Supplementary Materials

The following supporting information can be downloaded at: https://www.mdpi.com/article/10.3390/jcm15114245/s1, Supplementary Table S1: PRISMA 2020 checklist; Supplementary Table S2: Search strategy; Supplementary Table S3: GRADE certainty of evidence; Supplementary Figure S1: Forest plot of subgroup analysis for weaning success rate stratified by the invasiveness of the intervention; Supplementary Figure S2: Forest plot of subgroup analysis for weaning success rate stratified by the baseline duration of mechanical ventilation; Supplementary Figure S3: Sensitivity analysis for the duration of mechanical ventilation using a random-effects model; Supplementary Figure S4: Sensitivity analysis for maximal inspiratory pressure using a random-effects model; Supplementary Figure S5: Sensitivity analysis for ICU length of stay using a random-effects model; Supplementary Figure S6: Sensitivity analysis for the tracheostomy rate using a random-effects model.

## Abbreviations

The following abbreviations are used in this manuscript:

PNS	phrenic nerve stimulation.
ICU	intensive care unit.
RCT	randomized controlled trial.
RoB 2	Risk of Bias tool version 2.
MV	mechanical ventilation.
ARDS	acute respiratory distress syndrome.
VIDD	ventilator-induced diaphragmatic dysfunction.
PMV	prolonged mechanical ventilation.
VAP	ventilator-associated pneumonia.
SCI	spinal cord injury.
CSA	central sleep apnea.
EDP	external diaphragmatic pacing.
TEDS	transcutaneous electrical diaphragmatic stimulation.
TTDN	temporary transvenous diaphragm neurostimulation.
PRISMA	Preferred Reporting Items for Systematic reviews and Meta-analyses.
DMV	duration of mechanical ventilation.
MIP	maximal inspiratory pressure.
DTF	diaphragm thickening fraction.
ILOS	ICU length of stay.
RSBI	rapid shallow breathing index.
MeSH	Medical Subject Headings.
OR	odds ratio.
CI	confidence interval.
MD	mean difference.
REM	random-effects model.
FEM	fixed-effects model.
ICU-AW	ICU-acquired weakness.

## References

[1] Pham T., Heunks L., Bellani G., Madotto F., Aragao I., Beduneau G., Goligher E.C., Grasselli G., Laake J.H., Mancebo J. (2023). Weaning from Mechanical Ventilation in Intensive Care Units across 50 Countries (WEAN SAFE): A Multicentre, Prospective, Observational Cohort Study. Lancet Respir. Med..

[2] Qadir N., Sahetya S., Munshi L., Summers C., Abrams D., Beitler J., Bellani G., Brower R.G., Burry L., Chen J.-T. (2024). An Update on Management of Adult Patients with Acute Respiratory Distress Syndrome: An Official American Thoracic Society Clinical Practice Guideline. Am. J. Respir. Crit. Care Med..

[3] Rubulotta F., Blanch Torra L., Naidoo K.D., Aboumarie H.S., Mathivha L.R., Asiri A.Y., Sarlabous Uranga L., Soussi S. (2024). Mechanical Ventilation, Past, Present, and Future. Anesth. Analg..

[4] Fu W., Guan L., Liu Q., Xie Z., You J., Chen R. (2025). Ventilator-Induced Diaphragmatic Dysfunction: Pathophysiology, Monitoring and Advances in Potential Treatment and Prevention. Eur. Respir. Rev..

[5] Goligher E.C., Dres M., Fan E., Rubenfeld G.D., Scales D.C., Herridge M.S., Vorona S., Sklar M.C., Rittayamai N., Lanys A. (2018). Mechanical Ventilation-Induced Diaphragm Atrophy Strongly Impacts Clinical Outcomes. Am. J. Respir. Crit. Care Med..

[6] Huang H.-Y., Huang C.-Y., Li L.-F. (2022). Prolonged Mechanical Ventilation: Outcomes and Management. J. Clin. Med..

[7] Stivi T., Padawer D., Dirini N., Nachshon A., Batzofin B.M., Ledot S. (2024). Using Artificial Intelligence to Predict Mechanical Ventilation Weaning Success in Patients with Respiratory Failure, Including Those with Acute Respiratory Distress Syndrome. J. Clin. Med..

[8] van den Berg M.J.W., Heunks L., Doorduin J. (2025). Advances in Achieving Lung and Diaphragm-Protective Ventilation. Curr. Opin. Crit. Care.

[9] Farley C., Oliveira A., Brooks D., Newman A.N.L. (2026). The Effects of Inspiratory Muscle Training in Critically Ill Adults: A Systematic Review and Meta-Analysis. J. Intensive Care Med..

[10] Panelli A., Grimm A.M., Krause S., Verfuß M.A., Ulm B., Grunow J.J., Bartels H.G., Carbon N.M., Niederhauser T., Weber-Carstens S. (2024). Noninvasive Electromagnetic Phrenic Nerve Stimulation in Critically Ill Patients: A Feasibility Study. Chest.

[11] Rohrs E.C., Reynolds S., Dres M. (2025). Diaphragm Neurostimulation in Mechanical Ventilation: Current Status and Future Prospects. Expert. Rev. Med. Devices.

[12] Etienne H., Morris I.S., Hermans G., Heunks L., Goligher E.C., Jaber S., Morelot-Panzini C., Assouad J., Gonzalez-Bermejo J., Papazian L. (2023). Diaphragm Neurostimulation Assisted Ventilation in Critically Ill Patients. Am. J. Respir. Crit. Care Med..

[13] Costanzo M.R., Ponikowski P., Javaheri S., Augostini R., Goldberg L., Holcomb R., Kao A., Khayat R.N., Oldenburg O., Stellbrink C. (2016). Transvenous Neurostimulation for Central Sleep Apnoea: A Randomised Controlled Trial. Lancet.

[14] Wijkstra P.J., van der Aa H., Hofker H.S., Curto F., Giacomini M., Stagni G., Dura Agullo M.A., Curià Casanoves F.X., Benito-Penalva J., Martinez-Barenys C. (2022). Diaphragm Pacing in Patients with Spinal Cord Injury: A European Experience. Respiration.

[15] Dres M., Demoule A. (2018). Diaphragm Dysfunction during Weaning from Mechanical Ventilation: An Underestimated Phenomenon with Clinical Implications. Crit. Care.

[16] Pellegrini M., Parfait M., Dres M. (2025). How to Protect the Diaphragm and the Lung with Diaphragm Neurostimulation. Curr. Opin. Crit. Care.

[17] Hsin Y.-F., Chen S.-H., Yu T.-J., Huang C.-C., Chen Y.-H. (2022). Effects of Transcutaneous Electrical Diaphragmatic Stimulation on Respiratory Function in Patients with Prolonged Mechanical Ventilation. Ann. Thorac. Med..

[18] Bao Q., Chen L., Chen X., Li T., Xie C., Zou Z., Huang C., Zhi Y., He Z. (2022). The Effects of External Diaphragmatic Pacing on Diaphragm Function and Weaning Outcomes of Critically Ill Patients with Mechanical Ventilation: A Prospective Randomized Study. Ann. Transl. Med..

[19] Medrinal C., Machefert M., Lamia B., Bonnevie T., Gravier F.-E., Hilfiker R., Prieur G., Combret Y. (2023). Transcutaneous Electrical Diaphragmatic Stimulation in Mechanically Ventilated Patients: A Randomised Study. Crit. Care.

[20] Olímpio Júnior H., Camilo G.B., Marques J.A., Xavier R.S., Santos C.E., Lopes A.J. (2024). Effects of Transcutaneous Electrical Diaphragmatic Stimulation in Critically Ill Elderly Patients: A Randomized Controlled Trial. Physiother. Theory Pract..

[21] Dres M., Ewert R., Conrad S.A., Ataya A., Shrager J., Mortaza S., Delamaire F., Nilius G., Heine A., Mehta N. (2026). Temporary Transvenous Diaphragm Neurostimulation for Weaning from Mechanical Ventilation (RESCUE-3): A Randomized Clinical Trial. Am. J. Respir. Crit. Care Med..

[22] Luni F.K., Daniels J., Link M.S., Joglar J.A., Zungsontiporn N., Wu R., Kaplish N., Malik S.A. (2020). Meta-Analysis of Usefulness of Phrenic Nerve Stimulation in Central Sleep Apnea. Am. J. Cardiol..

[23] Wang Y., Huang Y., Xia M., Salanitro M., Kraemer J.F., Toncar T., Fietze I., Schöbel C., Penzel T. (2023). Effect of Phrenic Nerve Stimulation on Patients with Central Sleep Apnea: A Meta-Analysis. Sleep Med. Rev..

[24] Arango-Cortes M.L., Giraldo-Cadavid L.F., Latorre Quintana M., Forero-Cubides J.D., Gonzalez-Bermejo J. (2024). Diaphragm Pacing Compared with Mechanical Ventilation in Patients with Chronic Respiratory Failure Caused by Diaphragmatic Dysfunction: A Systematic Review and Meta-Analysis. Expert Rev. Respir. Med..

[25] Tong S., Yang Y., Li Y., Liu L., Chang W. (2025). Consequences in Critically Ill Patients with Prolonged Mechanical Ventilation after Diaphragmatic Stimulation Techniques: A Systematic Review and Meta-Analysis. BMJ Open.

[26] Page M.J., McKenzie J.E., Bossuyt P.M., Boutron I., Hoffmann T.C., Mulrow C.D., Shamseer L., Tetzlaff J.M., Akl E.A., Brennan S.E. (2021). The PRISMA 2020 Statement: An Updated Guideline for Reporting Systematic Reviews. BMJ.

[27] Burns K.E.A., Rizvi L., Cook D.J., Lebovic G., Dodek P., Villar J., Slutsky A.S., Jones A., Kapadia F.N., Gattas D.J. (2021). Ventilator Weaning and Discontinuation Practices for Critically Ill Patients. JAMA.

[28] Medrinal C., Prieur G., Frenoy É., Robledo Quesada A., Poncet A., Bonnevie T., Gravier F.-E., Lamia B., Contal O. (2016). Respiratory Weakness after Mechanical Ventilation Is Associated with One-Year Mortality—A Prospective Study. Crit. Care.

[29] Tuinman P.R., Jonkman A.H., Dres M., Shi Z.-H., Goligher E.C., Goffi A., de Korte C., Demoule A., Heunks L. (2020). Respiratory Muscle Ultrasonography: Methodology, Basic and Advanced Principles and Clinical Applications in ICU and ED Patients-a Narrative Review. Intensive Care Med..

[30] Trivedi V., Chaudhuri D., Jinah R., Piticaru J., Agarwal A., Liu K., McArthur E., Sklar M.C., Friedrich J.O., Rochwerg B. (2022). The Usefulness of the Rapid Shallow Breathing Index in Predicting Successful Extubation: A Systematic Review and Meta-Analysis. Chest.

[31] Sterne J.A.C., Savović J., Page M.J., Elbers R.G., Blencowe N.S., Boutron I., Cates C.J., Cheng H.-Y., Corbett M.S., Eldridge S.M. (2019). RoB 2: A Revised Tool for Assessing Risk of Bias in Randomised Trials. BMJ.

[32] Guyatt G.H., Oxman A.D., Vist G.E., Kunz R., Falck-Ytter Y., Alonso-Coello P., Schünemann H.J. (2008). GRADE Working Group GRADE: An Emerging Consensus on Rating Quality of Evidence and Strength of Recommendations. BMJ.

[33] Wan X., Wang W., Liu J., Tong T. (2014). Estimating the Sample Mean and Standard Deviation from the Sample Size, Median, Range and/or Interquartile Range. BMC Med. Res. Methodol..

[34] Higgins J.P.T., Thompson S.G., Deeks J.J., Altman D.G. (2003). Measuring Inconsistency in Meta-Analyses. BMJ.

[35] Egger M., Davey Smith G., Schneider M., Minder C. (1997). Bias in Meta-Analysis Detected by a Simple, Graphical Test. BMJ.

[36] Garnacho-Montero J., Amaya-Villar R., García-Garmendía J.L., Madrazo-Osuna J., Ortiz-Leyba C. (2005). Effect of Critical Illness Polyneuropathy on the Withdrawal from Mechanical Ventilation and the Length of Stay in Septic Patients. Crit. Care Med..

[37] Reynolds S., Ebner A., Meffen T., Thakkar V., Gani M., Taylor K., Clark L., Sadarangani G., Meyyappan R., Sandoval R. (2017). Diaphragm Activation in Ventilated Patients Using a Novel Transvenous Phrenic Nerve Pacing Catheter. Crit. Care Med..

[38] Rohrs E.C., Bassi T.G., Fernandez K.C., Ornowska M., Nicholas M., Wittmann J.C., Reynolds S.C. (2021). Diaphragm Neurostimulation during Mechanical Ventilation Reduces Atelectasis and Transpulmonary Plateau Pressure, Preserving Lung Homogeneity and PaO_2_/FiO_2_. J. Appl. Physiol..

[39] Wu Y., Wang S., Zhang J., Wang Y., Zhong J., Wang Y. (2024). Effects of Diaphragm Electrical Stimulation in Treating Respiratory Dysfunction on Mechanical Ventilation after Intracerebral Hemorrhage: A Single-Center Retrospective Study. Medicine.

[40] Fernandez K.C., Rohrs E.C., Bassi T.G., Ornowska M., Nicholas M., Gani M., Reynolds S.C. (2023). Transvenous Stimulation Yields Exposure-Dependent Protection from Ventilator-Induced Diaphragm Atrophy. J. Appl. Physiol..

[41] Ruan T. (2026). Recovery of Motor Functions and Cognitive Functions in Patients with Intensive Care Unit-Acquired Weakness: Methodological Considerations and Future Directions. J. Crit. Care.

[42] Forgiarini S.G.I., da Rosa D.P., Forgiarini L.F., Teixeira C., Andrade C.F., Forgiarini Junior L.A., Felix E.A., Friedman G. (2018). Evaluation of Systemic Inflammation in Patients Being Weaned from Mechanical Ventilation. Clinics.

[43] Torres-Castro R., Caicedo-Trujillo S., Gimeno-Santos E., Gutiérrez-Arias R., Alsina-Restoy X., Vasconcello-Castillo L., Seron P., Spruit M.A., Blanco I., Vilaró J. (2025). Effectiveness of Inspiratory Muscle Training in Patients with a Chronic Respiratory Disease: An Overview of Systematic Reviews. Front. Sports Act. Living.

[44] Dres M., de Abreu M.G., Merdji H., Müller-Redetzky H., Dellweg D., Randerath W.J., Mortaza S., Jung B., Bruells C., Moerer O. (2022). Randomized Clinical Study of Temporary Transvenous Phrenic Nerve Stimulation in Difficult-to-Wean Patients. Am. J. Respir. Crit. Care Med..

[45] Parada-Gereda H.M., Tibaduiza A.L., Rico-Mendoza A., Molano-Franco D., Nieto V.H., Arias-Ortiz W.A., Perez-Terán P., Masclans J.R. (2023). Effectiveness of Diaphragmatic Ultrasound as a Predictor of Successful Weaning from Mechanical Ventilation: A Systematic Review and Meta-Analysis. Crit. Care.

[46] Shen Y., Zhang H., Wang L., Song X., Wang X., Cao A. (2025). Effect of transcutaneous phrenic nerve stimulation in preventing ventilator-induced diaphragmatic dysfunction in invasive mechanically ventilated patients. Zhonghua Wei Zhong Bing Ji Jiu Yi Xue.

[47] Virolle S., Duceau B., Morawiec E., Fossé Q., Nierat M.-C., Parfait M., Decavèle M., Demoule A., Delemazure J., Dres M. (2024). Contribution and Evolution of Respiratory Muscles Function in Weaning Outcome of Ventilator-Dependent Patients. Crit. Care.

[48] Dres M., Goligher E.C., Dubé B.-P., Morawiec E., Dangers L., Reuter D., Mayaux J., Similowski T., Demoule A. (2018). Diaphragm Function and Weaning from Mechanical Ventilation: An Ultrasound and Phrenic Nerve Stimulation Clinical Study. Ann. Intensive Care.

[49] Papazian L., Klompas M., Luyt C.-E. (2020). Ventilator-Associated Pneumonia in Adults: A Narrative Review. Intensive Care Med..

[50] Guo Y., Wang F., Ma S., Mao Z., Zhao S., Sui L., Jiao C., Lu R., Zhu X., Pan X. (2025). Relationship between Atherogenic Index of Plasma and Length of Stay in Critically Ill Patients with Atherosclerotic Cardiovascular Disease: A Retrospective Cohort Study and Predictive Modeling Based on Machine Learning. Cardiovasc. Diabetol..

[51] Voiriot G., Oualha M., Pierre A., Salmon-Gandonnière C., Gaudet A., Jouan Y., Kallel H., Radermacher P., Vodovar D., Sarton B. (2022). Chronic Critical Illness and Post-Intensive Care Syndrome: From Pathophysiology to Clinical Challenges. Ann. Intensive Care.

[52] Xie D., Xu H., Wang F., Wen W., Dong B. (2025). Diagnostic Accuracy of Rapid Shallow Breathing Index Based on Diaphragm Ultrasound Predicting Successful Weaning from Mechanical Ventilation: A Systematic Review and Meta-Analysis. Intensive Crit. Care Nurs..

[53] Vaporidi K., Akoumianaki E., Telias I., Goligher E.C., Brochard L., Georgopoulos D. (2020). Respiratory Drive in Critically Ill Patients. Pathophysiology and Clinical Implications. Am. J. Respir. Crit. Care Med..

[54] Calderone A., Filoni S., De Luca R., Corallo F., Calapai R., Mirabile A., Caminiti F., Conti-Nibali V., Quartarone A., Calabrò R.S. (2025). Predictive Factors of Successful Decannulation in Tracheostomy Patients: A Scoping Review. J. Clin. Med..

[55] Dubé B.-P., Dres M., Mayaux J., Demiri S., Similowski T., Demoule A. (2017). Ultrasound Evaluation of Diaphragm Function in Mechanically Ventilated Patients: Comparison to Phrenic Stimulation and Prognostic Implications. Thorax.

[56] Laghi F., Shaikh H., Littleton S.W., Morales D., Jubran A., Tobin M.J. (2020). Inhibition of Central Activation of the Diaphragm: A Mechanism of Weaning Failure. J. Appl. Physiol..

[57] Greendyk R.A., Abrams D., Agerstrand C. (2026). Weaning from Venovenous Extracorporeal Membrane Oxygenation for Acute Respiratory Failure: Challenges and Opportunities. Curr. Opin. Crit. Care.

[58] Jonkman A.H., Rauseo M., Carteaux G., Telias I., Sklar M.C., Heunks L., Brochard L.J. (2020). Proportional Modes of Ventilation: Technology to Assist Physiology. Intensive Care Med..

[59] Scaramuzzo G., Pavlovsky B., Adler A., Baccinelli W., Bodor D.L., Damiani L.F., Franchineau G., Francovich J., Frerichs I., Giralt J.A.S. (2024). Electrical Impedance Tomography Monitoring in Adult ICU Patients: State-of-the-Art, Recommendations for Standardized Acquisition, Processing, and Clinical Use, and Future Directions. Crit. Care.

