# Supplementary material for: Effects of Phrenic Nerve Stimulation in Mechanically Ventilated Patients: A Systematic Review and Meta-Analysis of Randomized Controlled Trials
Source: J Clin Med. 2026 May 30;15(11):4245. doi: 10.3390/jcm15114245 (PMC13258714; doi:10.3390/jcm15114245)

Supplementary Figure S1. Forest plot of subgroup analysis for weaning success rate stratified by the invasiveness of the intervention. PNS = phrenic nerve stimulation; M-H = Mantel-Haenszel method; CI = confidence interval; OR = odds ratio; TEDS = transcutaneous electrical diaphragmatic stimulation; EDP = external diaphragmatic pacing; TTDN = temporary transvenous diaphragm neurostimulation.

	PNS		Control			Odds Ratio
Study or Subgroup	Events	Total	Events	Total	Weight	M-H, Random, 95% CI
**1.2.1 invasive**						
Dres 2026	71	102	69	114	33.7%	1.49 [0.85, 2.63]
**Subtotal (95% CI)**		**102**		**114**	**33.7%**	**1.49 [0.85, 2.63]**
Total events	71		69			
Heterogeneity: Not applicable						
Test for overall effect: Z = 1.39 (P = 0.16)						
**1.2.2 non-invasive**						
Bao 2022	17	27	3	24	21.9%	11.90 [2.82, 50.23]
Hsin 2022	27	29	20	30	19.6%	6.75 [1.33, 34.26]
Medrinal 2023	17	24	17	25	24.8%	1.14 [0.34, 3.86]
Olímpio 2024	23	23	21	21		Not estimable
**Subtotal (95% CI)**		**103**		**100**	**66.3%**	**4.27 [0.96, 19.06]**
Total events	84		61			
Heterogeneity: Tau² = 1.22; Chi² = 6.65, df = 2 (P = 0.04); I² = 70%						
Test for overall effect: Z = 1.90 (P = 0.06)						
**Total (95% CI)**		**205**		**214**	**100.0%**	**2.96 [1.04, 8.40]**
Total events	155		130			
Heterogeneity: Tau² = 0.76; Chi² = 9.95, df = 3 (P = 0.02); I² = 70%						
Test for overall effect: Z = 2.04 (P = 0.04)						
Test for subgroup differences: Chi² = 1.66, df = 1 (P = 0.20), I² = 39.8%						

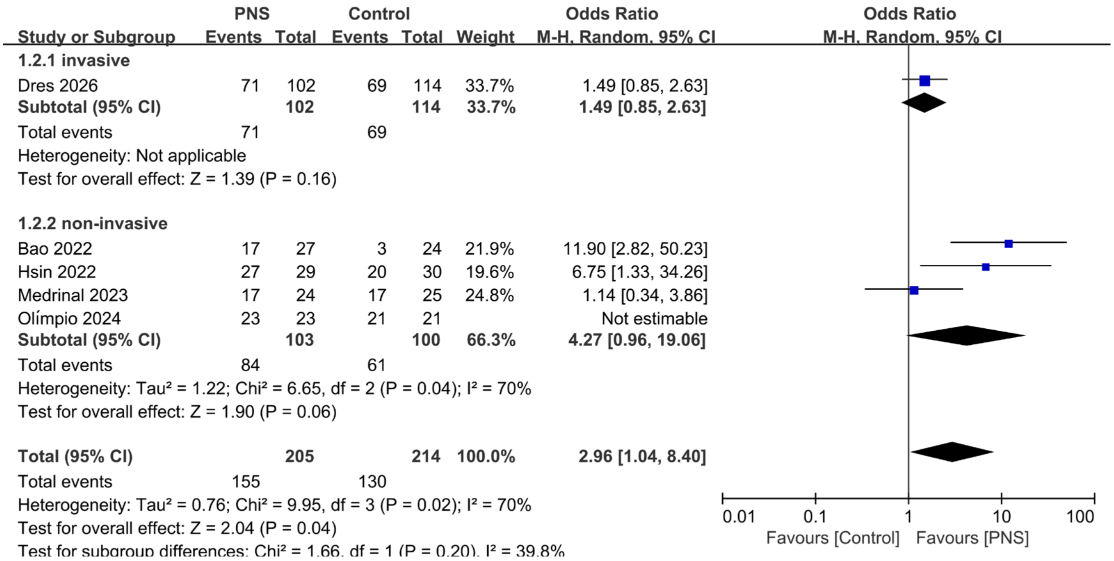

Supplementary Figure S2. Forest plot of subgroup analysis for weaning success rate stratified by the baseline duration of mechanical ventilation. PNS = phrenic nerve stimulation; M-H = Mantel-Haenszel method; CI = confidence interval; OR = odds ratio.

	PNS		Control			Odds Ratio
Study or Subgroup	Events	Total	Events	Total	Weight	M-H, Random, 95% CI
**1.3.1 MV ⩾ 24 hours**						
Medrinal 2023	17	24	17	25	24.8%	1.14 [0.34, 3.86]
Olímpio 2024	23	23	21	21		Not estimable
**Subtotal (95% CI)**		**47**		**46**	**24.8%**	**1.14 [0.34, 3.86]**
Total events	40		38			
Heterogeneity: Not applicable						
Test for overall effect: Z = 0.22 (P = 0.83)						
**1.3.2 prolonged mechanical ventilation**						
Bao 2022	17	27	3	24	21.9%	11.90 [2.82, 50.23]
Dres 2026	71	102	69	114	33.7%	1.49 [0.85, 2.63]
Hsin 2022	27	29	20	30	19.6%	6.75 [1.33, 34.26]
**Subtotal (95% CI)**		**158**		**168**	**75.2%**	**4.32 [1.01, 18.52]**
Total events	115		92			
Heterogeneity: Tau² = 1.26; Chi² = 8.98, df = 2 (P = 0.01); I² = 78%						
Test for overall effect: Z = 1.97 (P = 0.05)						
**Total (95% CI)**		**205**		**214**	**100.0%**	**2.96 [1.04, 8.40]**
Total events	155		130			
Heterogeneity: Tau² = 0.76; Chi² = 9.95, df = 3 (P = 0.02); I² = 70%						
Test for overall effect: Z = 2.04 (P = 0.04)						
Test for subgroup differences: Chi² = 1.89, df = 1 (P = 0.17), I² = 47.1%						

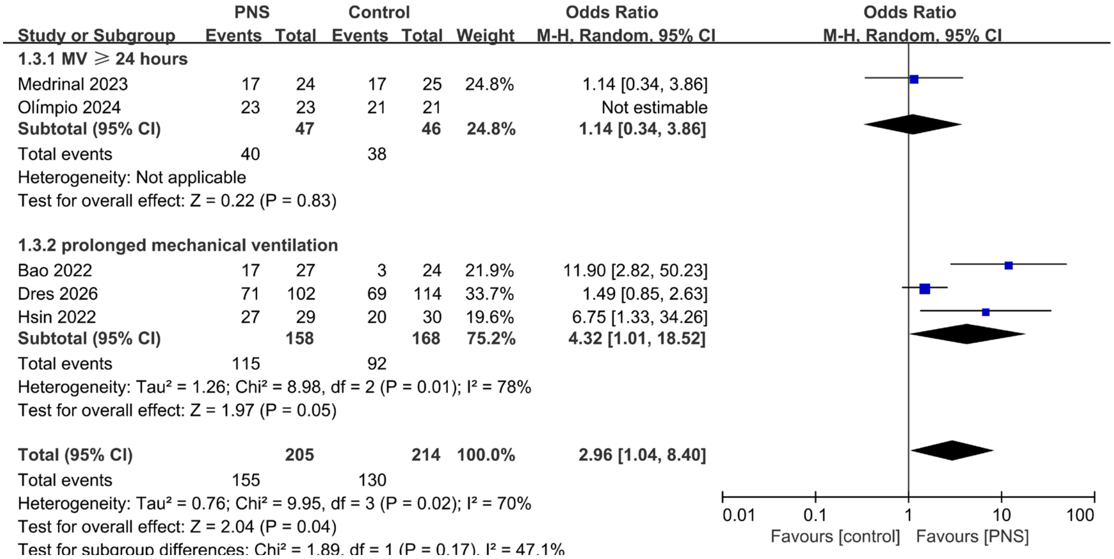

Supplementary Figure S3. Sensitivity analysis for the duration of mechanical ventilation using a random-effects model. PNS = phrenic nerve stimulation; MD = mean difference; CI = confidence interval; DMV = duration of mechanical ventilation.

	PNS			Control				Mean Difference
Study or Subgroup	Mean	SD	Total	Mean	SD	Total	Weight	IV, Random, 95% CI
Bao 2022	14.14	9.39	27	18.48	12.01	24	4.6%	-4.34 [-10.31, 1.63]
Dres 2026	16.17	17.3	102	18	16.52	114	7.9%	-1.83 [-6.35, 2.69]
Hsin 2022	14.5	10.9	29	19.5	14.2	30	3.9%	-5.00 [-11.45, 1.45]
Medrinal 2023	7.82	4.28	30	9.03	6.61	31	20.9%	-1.21 [-4.00, 1.58]
Olímpio 2024	6.28	2.68	23	9.21	2.76	21	62.7%	-2.93 [-4.54, -1.32]
**Total (95% CI)**			**211**			**220**	**100.0%**	**-2.63 [-3.90, -1.35]**

Heterogeneity: Tau² = 0.00; Chi² = 2.09, df = 4 (P = 0.72); I² = 0%
Test for overall effect: Z = 4.04 (P < 0.0001)

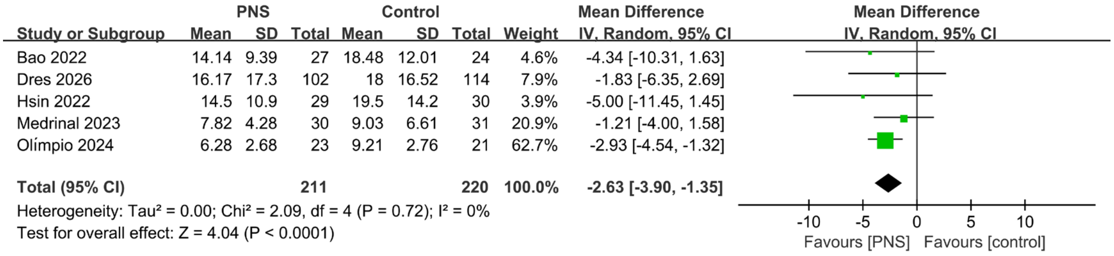

Supplementary Figure S4. Sensitivity analysis for maximal inspiratory pressure using a random-effects model. PNS = phrenic nerve stimulation; MD = mean difference; CI = confidence interval; MIP = maximal inspiratory pressure.

	PNS			Control				Mean Difference
Study or Subgroup	Mean	SD	Total	Mean	SD	Total	Weight	IV, Random, 95% CI
Dres 2026	12.1	17.75	102	9	14.34	114	18.2%	3.10 [-1.24, 7.44]
Hsin 2022	25.71	18.71	29	25.43	8.56	30	6.1%	0.28 [-7.19, 7.75]
Medrinal 2023	35.57	11.9	30	29.71	11.15	31	10.2%	5.86 [0.07, 11.65]
Olímpio 2024	22.04	3.41	23	19.34	4.23	21	65.5%	2.70 [0.42, 4.98]
**Total (95% CI)**			**184**			**196**	**100.0%**	**2.95 [1.10, 4.79]**

Heterogeneity: Tau² = 0.00; Chi² = 1.51, df = 3 (P = 0.68); I² = 0%

Test for overall effect: Z = 3.12 (P = 0.002)

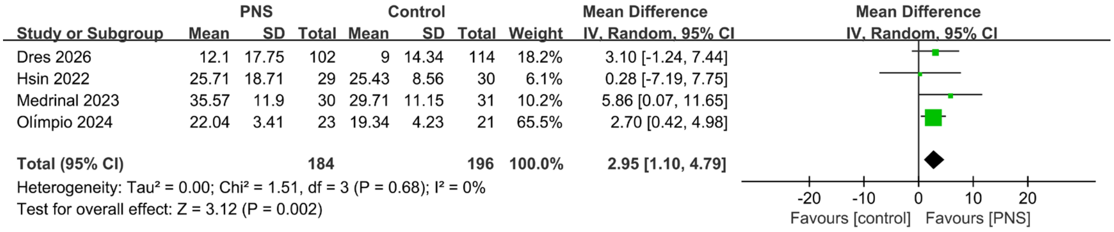

Supplementary Figure S5. Sensitivity analysis for ICU length of stay using a random-effects model. PNS = phrenic nerve stimulation; MD = mean difference; CI = confidence interval; ICU = intensive care unit; ILOS = ICU length of stay.

	PNS			Control				Mean Difference	Mean Difference
**Study or Subgroup**	**Mean**	**SD**	**Total**	**Mean**	**SD**	**Total**	**Weight**	**IV, Random, 95% CI**	**IV, Random, 95% CI**
Bao 2022	16.86	11.74	27	20.93	11.03	24	14.5%	-4.07 [-10.32, 2.18]	
Dres 2026	22.54	12.78	102	22.95	14.42	114	43.0%	-0.41 [-4.04, 3.22]	
Hsin 2022	20.8	10	29	22.7	8.6	30	24.9%	-1.90 [-6.67, 2.87]	
Medrinal 2023	14.89	9.52	30	18.21	12.83	31	17.7%	-3.32 [-8.98, 2.34]	
**Total (95% CI)**			**188**			**199**	**100.0%**	**-1.82 [-4.20, 0.55]**	

Heterogeneity: Tau² = 0.00; Chi² = 1.35, df = 3 (P = 0.72); I² = 0%

Test for overall effect: Z = 1.50 (P = 0.13)

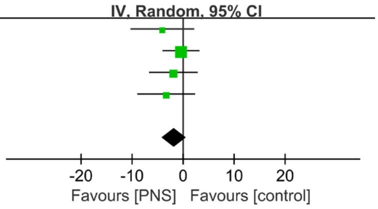

Supplementary Figure S6. Sensitivity analysis for the tracheostomy rate using a random-effects model. PNS = phrenic nerve stimulation; M-H = Mantel-Haenszel method; CI = confidence interval; OR = odds ratio.

	PNS		Control			Odds Ratio
**Study or Subgroup**	**Events**	**Total**	**Events**	**Total**	**Weight**	**M-H, Random, 95% CI**
Dres 2026	5	39	7	38	57.2%	0.65 [0.19, 2.27]
Medrinal 2023	5	27	4	26	42.8%	1.25 [0.30, 5.28]
**Total (95% CI)**		**66**		**64**	**100.0%**	**0.86 [0.34, 2.21]**
Total events	10		11			

Heterogeneity: Tau² = 0.00; Chi² = 0.45, df = 1 (P = 0.50); I² = 0%
Test for overall effect: Z = 0.31 (P = 0.76)

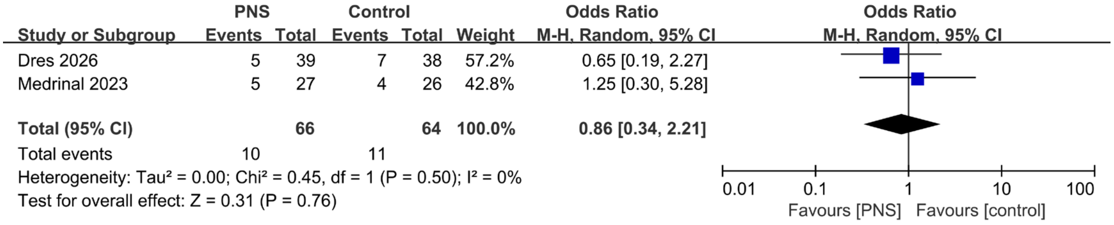

Supplement: Supplementary file 1 [file jcm-15-04245-s001.zip › Supplementary Figure.pdf]
