# Supplementary material for: Effects of Phrenic Nerve Stimulation in Mechanically Ventilated Patients: A Systematic Review and Meta-Analysis of Randomized Controlled Trials
Source: J Clin Med. 2026 May 30;15(11):4245. doi: 10.3390/jcm15114245 (PMC13258714; doi:10.3390/jcm15114245)
Supplement: Supplementary file 1 [file jcm-15-04245-s001.zip › Supplementary Table S2 and S3.pdf]

Supplementary Table S2. Search strategy

	**Search strategy（PubMed）**	**Results**
1	Electric Stimulation[MeSH Terms]	131,651
2	"Stimulation, Electric"[Title/Abstract] OR "Electric Stimulations"[Title/Abstract] OR "Stimulations, Electric"[Title/Abstract] OR "Electrical Stimulation"[Title/Abstract] OR "Electrical Stimulations"[Title/Abstract] OR "Stimulation, Electrical"[Title/Abstract] OR "Stimulations, Electrical"[Title/Abstract]	55,187
3	(Electric Stimulation[MeSH Terms]) OR ("Stimulation, Electric"[Title/Abstract] OR "Electric Stimulations"[Title/Abstract] OR "Stimulations, Electric"[Title/Abstract] OR "Electrical Stimulation"[Title/Abstract] OR "Electrical Stimulations"[Title/Abstract] OR "Stimulation, Electrical"[Title/Abstract] OR "Stimulations, Electrical"[Title/Abstract])	159,747
4	Phrenic Nerve[MeSH Terms]	7,089
5	"Nerve, Phrenic"[Title/Abstract] OR "Nerves, Phrenic"[Title/Abstract] OR "Phrenic Nerves"[Title/Abstract] OR "Phrenic nerves stimulation"[Title/Abstract]	966
6	Diaphragm[MeSH Terms]	37,242
7	((((((Diaphragms[Title/Abstract]) OR (Respiratory Diaphragm[Title/Abstract])) OR (Diaphragm,Respiratory[Title/Abstract])) OR (Diaphragms,Respiratory[Title/Abstract])) OR (Respiratory Diaphragms[Title/Abstract])) OR (Diaphragm pacing[Title/Abstract])) OR (Diaphragm stimulation[Title/Abstract])	9,832
8	(((Phrenic Nerve[MeSH Terms]) OR ("Nerve, Phrenic"[Title/Abstract] OR "Nerves, Phrenic"[Title/Abstract] OR "Phrenic Nerves"[Title/Abstract] OR "Phrenic nerves stimulation"[Title/Abstract])) OR (Diaphragm[MeSH Terms])) OR (((((((Diaphragms[Title/Abstract]) OR (Respiratory Diaphragm[Title/Abstract])) OR (Diaphragm,Respiratory[Title/Abstract])) OR (Diaphragms,Respiratory[Title/Abstract])) OR (Respiratory Diaphragms[Title/Abstract])) OR (Diaphragm pacing[Title/Abstract])) OR (Diaphragm stimulation[Title/Abstract]))	46,558
9	Respiration, Artificial[MeSH Terms]	94,630
10	((((((Artificial Respiration[Title/Abstract]) OR (Artificial Respirations[Title/Abstract])) OR (Respirations, Artificial[Title/Abstract])) OR (Ventilation, Mechanical[Title/Abstract])) OR (Mechanical Ventilations[Title/Abstract])) OR (Ventilations, Mechanical[Title/Abstract])) OR (Mechanical Ventilation[Title/Abstract])	75,071
11	(Respiration, Artificial[MeSH Terms]) OR (((((((Artificial Respiration[Title/Abstract]) OR (Artificial Respirations[Title/Abstract])) OR (Respirations, Artificial[Title/Abstract])) OR (Ventilation, Mechanical[Title/Abstract])) OR (Mechanical Ventilations[Title/Abstract])) OR (Ventilations, Mechanical[Title/Abstract])) OR (Mechanical Ventilation[Title/Abstract]))	137,008
12	(((Electric Stimulation[MeSH Terms]) OR ("Stimulation, Electric"[Title/Abstract] OR "Electric Stimulations"[Title/Abstract] OR "Stimulations, Electric"[Title/Abstract] OR "Electrical Stimulation"[Title/Abstract] OR "Electrical Stimulations"[Title/Abstract] OR "Stimulation, Electrical"[Title/Abstract] OR "Stimulations, Electrical"[Title/Abstract])) AND ((((Phrenic Nerve[MeSH Terms]) OR ("Nerve, Phrenic"[Title/Abstract] OR "Nerves, Phrenic"[Title/Abstract] OR "Phrenic Nerves"[Title/Abstract] OR "Phrenic nerves stimulation"[Title/Abstract])) OR (Diaphragm[MeSH Terms])) OR (((((((Diaphragms[Title/Abstract]) OR (Respiratory Diaphragm[Title/Abstract])) OR (Diaphragm,Respiratory[Title/Abstract])) OR	202

(Diaphragms,Respiratory[Title/Abstract])) OR (Respiratory Diaphragms[Title/Abstract])) OR (Diaphragm pacing[Title/Abstract])) OR (Diaphragm stimulation[Title/Abstract])))) AND ((Respiration, Artificial[MeSH Terms]) OR (((((((Artificial Respiration[Title/Abstract]) OR (Artificial Respirations[Title/Abstract])) OR (Respirations, Artificial[Title/Abstract])) OR (Ventilation, Mechanical[Title/Abstract])) OR (Mechanical Ventilations[Title/Abstract])) OR (Ventilations, Mechanical[Title/Abstract])) OR (Mechanical Ventilation[Title/Abstract])))

	**Search strategy（Embase）**	**Results**
#1	'electrostimulation'/exp OR 'electrostimulation'	104384
#2	'electric field stimulation'/exp OR 'electric field stimulation' OR 'electric stimulation'/exp OR 'electric stimulation' OR 'electrical stimulation'/exp OR 'electrical stimulation' OR 'electro stimulation'/exp OR 'electro stimulation' OR 'electrostimulus'/exp OR 'electrostimulus' OR 'galvanostimulation'/exp OR 'galvanostimulation' OR 'stimulation, electric'/exp OR 'stimulation, electric' OR 'electrostimulation'/exp OR 'electrostimulation'	139794
#3	#1 OR #2	139794
#4	phrenic AND nerve	17229
#5	'diaphragm innervation' OR 'diaphragm nerve supply' OR 'nerve, phrenic' OR 'nervus phrenicus' OR 'phrenic nerve' OR 'phrenic nerves stimulation'	16501
#6	diaphragm	84080
#7	'diaphragm muscle' OR 'diaphragm muscle fiber' OR 'diaphragma' OR 'diaphragmatic muscle' OR 'diaphragm' OR 'diaphragm stimulation' OR 'diaphragm pacing'	84582
#8	#4 OR #5 OR #6 OR #7	95396
#9	artificial AND ventilation	224303
#10	'artificial respiration' OR 'artificial respiratory support' OR 'artificial ventilatory support' OR 'controlled respiration' OR 'controlled ventilation' OR 'mechanical respiration' OR 'mechanical ventilation' OR 'respiration, artificial' OR 'ventilation, artificial' OR 'artificial ventilation'	248382
#11	#9 OR #10	251713
#12	#3 AND #8 AND #11	316

	**Search strategy（Cochrane）**	**Results**
#1	MeSH descriptor: [Electric Stimulation] explode all trees	2605
#2	('Stimulation, Electric' OR 'Electric Stimulations' OR 'Stimulations, Electric' OR 'Electrical Stimulation' OR 'Electrical Stimulations' OR 'Stimulation, Electrical' OR 'Stimulations, Electrical'):ti,ab,kw	18363
#3	#1 OR #2	18517
#4	MeSH descriptor: [Phrenic Nerve] explode all trees	88
#5	('Nerve, Phrenic' OR 'Nerves, Phrenic' OR 'Phrenic Nerves' OR 'Phrenic nerves stimulation'):ti,ab,kw	716
#6	MeSH descriptor: [Diaphragm] explode all trees	410
#7	('Diaphragms' OR 'Respiratory Diaphragm' OR 'Diaphragm,Respiratory' OR 'Diaphragms,Respiratory' OR 'Respiratory Diaphragms' OR 'Diaphragm pacing' OR 'Diaphragm stimulation'):ti,ab,kw	1689
#8	#4 OR #5 OR #6 OR #7	2350
#9	MeSH descriptor: [Respiration, Artificial] explode all trees	9358
#10	('Artificial Respiration' OR 'Artificial Respirations' OR 'Respirations, Artificial' OR 'Ventilation, Mechanical' OR 'Mechanical Ventilations' OR 'Ventilations, Mechanical' OR 'Mechanical Ventilation'):ti,ab,kw	18886

#11	#9 OR #10	22277
#12	#3 AND #8 AND #11	52

	**Search strategy（Web of science）**	**Results**
1	TS=(Electric Stimulation OR Stimulation, Electric OR Electric Stimulations OR Stimulations, Electric OR Electrical Stimulation OR Electrical Stimulations OR Stimulation, Electrical OR Stimulations, Electrical)	103322
2	TS=(Phrenic Nerve OR Nerve, Phrenic OR Nerves, Phrenic OR Phrenic Nerves OR Phrenic nerves stimulation)	6995
3	TS=(Diaphragm OR Diaphragms OR Respiratory Diaphragm OR Diaphragm,Respiratory OR Diaphragms,Respiratory OR Respiratory Diaphragms OR Diaphragm pacing OR Diaphragm stimulation)	42747
4	TS=(Respiration, Artificial OR Artificial Respiration OR Artificial Respirations OR Respirations, Artificial OR Ventilation, Mechanical OR Mechanical Ventilations OR Ventilations, Mechanical OR Mechanical Ventilation)	83817
5	#2 OR #3	47533
6	#1 AND #5 AND #4	154

Supplementary Table S3. GRADE certainty of evidence

Certainty assessment						№ of patients		Effect		Certainty	Importance
**№ of studies**	**Study design**	**Risk of bias**	**Inconsistency**	**Indirectness**	**Imprecision**	**Other considerations**	**PNS**	**Control**	**Relative (95% CI)**	**Absolute (95% CI)**		
**Weaning success rate**											
5	randomised trials	not serious	serious[a]	not serious	serious[b]	none	155/205 (75.6%)	130/214 (60.7%)	**OR 2.96** (1.04 to 8.40)	**213 more per 1,000** (from 9 more to 321 more)	⊕⊕◯◯ Low	CRITICAL
**Duration of mechanical ventilation**											
5	randomised trials	not serious	not serious	not serious	not serious	none	211	220	-	MD **2.63 lower** (3.9 lower to 1.35 lower)	⊕⊕⊕⊕ High	CRITICAL
**Maximal inspiratory pressure**											
4	randomised trials	not serious	not serious	not serious	serious[c]	none	184	196	-	MD **2.95 higher** (1.1 higher to 4.79 higher)	⊕⊕⊕◯ Moderate	CRITICAL
**Diaphragm thickening fraction**											
3	randomised trials	not serious	serious[a]	not serious	serious[b]	none	80	76	-	MD **15.67 higher** (4.84 higher to 26.5 higher)	⊕⊕◯◯ Low	CRITICAL
**ICU length of stay**											
4	randomised trials	not serious	not serious	not serious	serious[d]	none	188	199	-	MD **1.82 lower** (4.2 lower to 0.55 higher)	⊕⊕⊕◯ Moderate	CRITICAL
**Rapid shallow breathing index**											
2	randomised trials	not serious	serious[a]	not serious	serious[e]	none	131	144	-	MD **11.45 lower** (30.06 lower to 7.15 higher)	⊕⊕◯◯ Low	CRITICAL
**Tracheostomy rate**											
2	randomised trials	not serious	not serious	not serious	very serious[f]	none	10/66 (15.2%)	11/64 (17.2%)	**OR 0.86** (0.34 to 2.19)	**20 fewer per 1,000** (from 106 fewer to 141 more)	⊕⊕◯◯ Low	CRITICAL

CI: confidence interval; **MD:** mean difference; **OR:** odds ratio

Explanations:

a. Downgraded one level due to substantial heterogeneity ($I^2 > 50\%$).

b. Downgraded one level due to insufficient sample size and wide 95% CI.

c. Downgraded one level because the effect estimate was small and its clinical importance remains uncertain, with possible measurement variability.

d. Downgraded one level due to no effect in 95% CI.

e. Downgraded one level due to no effect in 95% CI and insufficient sample size.

f. Downgraded two levels due to extremely insufficient sample size and no effect in 95% CI.
